# Novel complex of HAT protein TIP60 and nuclear receptor PXR promotes cell migration and adhesion

**DOI:** 10.1038/s41598-017-03783-w

**Published:** 2017-06-16

**Authors:** Karishma Bakshi, B. Ranjitha, Shraddha Dubey, Jaisri Jagannadham, Bharti Jaiswal, Ashish Gupta

**Affiliations:** Department of Life Sciences, Shiv Nadar University, Greater Noida, India

## Abstract

PXR is a member of nuclear receptor superfamily and a well-characterized mediator of xenobiotic metabolism. The classical mode of PXR activation involves its binding to appropriate ligand and subsequent heterodimerization with its partner RXR. However, various factors such as post-translational modifications and crosstalk with different cellular factors may also regulate the functional dynamics and behavior of PXR. In the present study, we have identified that TIP60, an essential lysine acetyltransferase protein interacts with unliganded PXR and together this complex promotes cell migration & adhesion. TIP60 utilizes its NR Box to interact with LBD region of PXR and acetylates PXR at lysine 170 to induce its intranuclear reorganization. Also, RXR is not required for TIP60-PXR complex formation and this complex does not induce ligand-dependent PXR target gene transactivation. Interestingly, we observed that PXR augments the catalytic activity of TIP60 for histones. This is the first report demonstrating the exclusive interaction of TIP60 with PXR and uncovers a potential role for the TIP60-PXR complex in cell migration and adhesion.

## Introduction

PXR is a well-recognized member of nuclear receptor (NR) superfamily and is known for its role in protecting the body against harmful accumulation of exogenous & endogenous chemicals by directing their metabolism and clearance^1–3^. PXR gets activated by binding of ligand which may alter its conformation and modulate its interaction with transcriptional coregulators. To exert its transcriptional function ligand-bound PXR binds to the response elements in the promoter region of its target genes as a heterodimer with Retinoid X receptor (RXR)^1, 4^. Although, it is primarily expressed in liver and intestine, varied expression level of PXR have been detected in many other tissues including ovaries, esophagus, breast, heart, brain and uterus^5^. The clinical relevance of differential tissue-specific expression pattern of PXR is not understood however this suggest tissue-specific or hitherto unknown diverse functions of PXR that are yet to be defined and fine-tuned. The modular structure of PXR is composed of a conserved DNA-binding domain (DBD) at N-terminus followed by a considerably short hinge region and a highly flexible and promiscuous ligand binding domain (LBD) at C-terminus. A ligand-dependent transactivation function 2 (AF-2) region located in the C-terminal region of LBD is essential for ligand-dependent interaction of PXR with transcriptional coregulator proteins.

Although PXR is prominently characterized as a xenobiotic sensor other functions of PXR have been discovered in recent years that can be extended to various physiological and pathophysiological conditions. Regulatory functions of PXR implicated in normal cellular physiology are mainly associated with homeostasis of glucose, lipids, steroid hormones and fat-soluble vitamins^3, 6^. Many metabolic disorders such as obesity, dyslipidemia, diabetes, bone disorders, hepatic steatosis and inflammatory bowel disease are linked directly or indirectly with anomalous expression and unwarranted activation or repression of PXR depending on the cellular microenvironment and tissue-type^7^. Number of studies have also demonstrated the implication of PXR in development and progression of many cancers^8, 9^. The capability of PXR to exert a wide range of physiological effects cannot be just attributed to its activation through ligands. Typically, the binding of cognate ligand is a requisite for PXR activation however substantial evidence suggests PXR activity can also be regulated by a variety of post-translational modifications (PTMs) like phosphorylation, SUMOylation and ubiquitination^10, 11^. Some reports have shown that PXR also gets acetylated *in vivo* and it is suggested that acetylation might regulate its functions^12, 13^. Recently, it is reported that dynamic acetylation and deacetylation of PXR at lysine 109 located in DBD region by P300 (HAT) & SIRT1 (HDAC) respectively modulate ligand-dependent transcriptional activity of PXR^14^. However, many more putative acetylation sites in PXR exists that remains to be assessed for their functional implications and also the enzymes responsible for catalyzing the transfer of acetyl group to these sites are yet to be identified.

Proteins are generally acetylated or deacetylated on lysine residues and the reaction is typically catalyzed by enzymes with histone acetyltransferase (HATs) or histone deacetyltransferase (HDACs) activity. TIP60 (Tat interactive protein) is one such lysine acetyl transferase protein of MYST family known to acetylate both histones & non-histone proteins^15, 16^. It is the only known HAT protein shown to be essential for cell survival as in Drosophila and human TIP60 homozygous knockout embryo does not survive^17, 18^. TIP60 serves as a transcriptional coregulator and plays important role in regulating transcription, DNA repair and apoptosis. Upon DNA damage, TIP60 activates the DNA repair pathway by acetylating ATM/ATR kinases and once repair is completed it helps in cessation of repair process by acetylating phospho-H2AX histones at the damaged sites^19–21^. In response to unrepairable DNA damage conditions, TIP60 can drive the equilibrium of cell towards apoptosis by acetylating p53^22, 23^. TIP60 promotes autophagy in the cell during serum deprivation condition by acetylating ULK1 kinase^24^.

As a nuclear receptor coregulator, TIP60 preferentially interact with and modulate class I NR signaling. TIP60 contains a single nuclear receptor box (NR Box) at its extreme C-terminus that facilitates its interaction with several class I NRs including androgen receptor (AR), estrogen receptor (ER) and progesterone receptor (PR)^25, 26^. For instance, TIP60 interacts with AR and modulate its intracellular dynamics and transcriptional activity^27, 28^. Similarly, TIP60 modulates transactivation function of PR, ERα and ERβ^25, 29, 30^. However, rare examples like PPARγ (a class II NR) and Rev-erbβ (an orphan receptor) are also shown to interact with TIP60^31, 32^.

Considering the significant involvement of TIP60 in functional regulation of different NRs we wanted to investigate whether TIP60 has any role to play in modulating the intracellular dynamics and function of class II NR PXR. In the present study, we have discovered that TIP60 and PXR interact with each other in a ligand-independent condition and form a unique complex independent of RXR. We also found that TIP60-PXR nuclear foci formation is dependent on lysine acetyl transferase activity of TIP60 to acetylate PXR and together this complex promotes cell migration & adhesion. Understanding how TIP60 could modulate subcellular dynamics of unliganded PXR and ascertaining the cellular functions regulated by this novel complex will lead us in expanding our knowledge about physiological and pathophysiological implications mediated through this complex.

## Results

### TIP60 interacts with unliganded PXR and modulates its intranuclear dynamics

**Figure 1:**
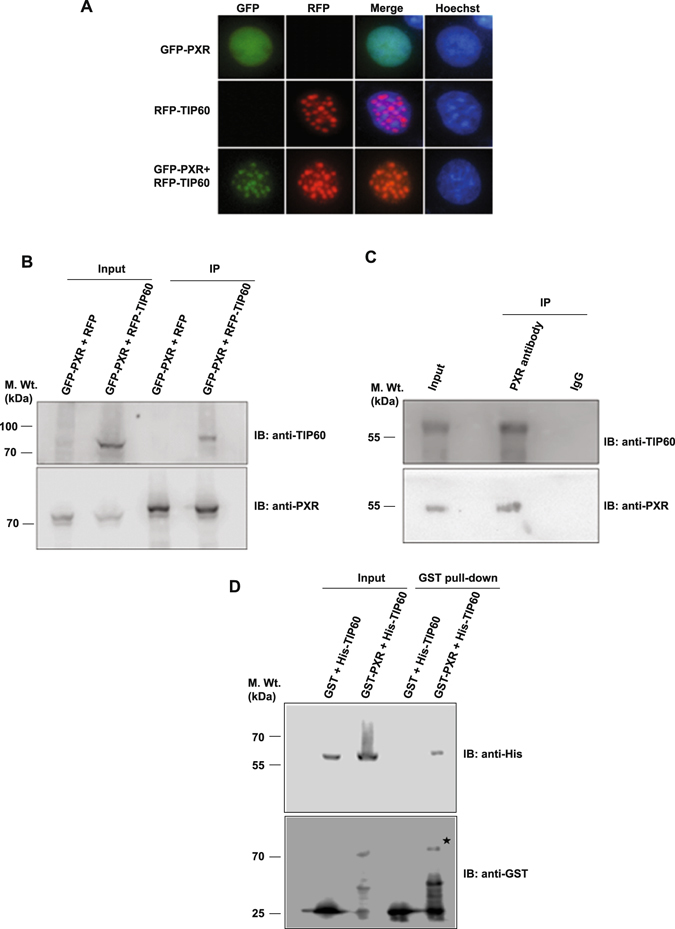
TIP60 modulates intranuclear dynamics of PXR and physically interacts with it. (**A**) Cos-1 cells were transfected with GFP-PXR and RFP-TIP60 plasmids alone or together and live cell imaging was performed to monitor their subcellular localization. (**B**) Cos-1 cells were transfected with GFP-PXR and RFP-TIP60 or RFP plasmids. Anti-PXR antibody was used for immunoprecipitation and immunoprecipitated proteins were resolved by SDS-PAGE. Immunoblotting was performed using anti-PXR or anti-TIP60 antibodies as indicated. Input lanes contain 10% of lysate used for immunoprecipitation. (**C**) To investigate endogenous interaction of TIP60 and PXR, endogenous PXR was immunoprecipitated from HepG2 cells using anti-PXR or control mouse IgG antibodies. Bound proteins were resolved by SDS-PAGE followed by immunoblotting with anti-PXR or anti-TIP60 antibodies. (**D**) TIP60 and PXR directly interact with each other. His-TIP60 and GST-PXR or GST alone were cotransformed and pull-down of glutathione-agarose-bound proteins was performed. Samples were resolved and immunoblotting was performed using anti-His or anti-GST antibodies. Asterisk indicates full-length band of GST-PXR protein. Full-length immunoblots are presented in Supplementary Figure S6A–C.

To examine the outcome of TIP60 and PXR coexpression on their respective intracellular localization, we generated fluorescent protein-tagged constructs of TIP60 and PXR and performed live cell imaging experiments. Cos-1 cells were transiently transfected with RFP-TIP60 and GFP-PXR plasmids either alone or in combination and fluorescence imaging was performed to monitor their intracellular localization. As shown in Fig. 1A, unliganded GFP-PXR when expressed alone showed nuclear localization with homogenous distribution pattern whereas RFP-TIP60 displayed nuclear localization with punctate foci formation. However, cells coexpressing both GFP-PXR and RFP-TIP60, showed a clear colocalization of both the proteins with a distinct nuclear punctate foci formation (Fig. 1A). A similar colocalization pattern of TIP60 and PXR was observed in various other cell lines and the observations were verified by counting (Supplementary Figure S1). Together these results show that subcellular localization of unliganded PXR is modulated by TIP60 and both the proteins colocalize inside the nucleus and form punctate foci.

Colocalization of TIP60 and PXR suggested a probable interaction between them and prompted us to investigate whether the two proteins interact with each other. To examine this, we performed *in vivo* immunoprecipitation assays. Cos-1 cells were transiently transfected with GFP-PXR along with RFP-TIP60 or RFP plasmids. Cell lysates were subjected to immunoprecipitation with PXR antibody followed by Western blot analysis of immunoprecipitated samples with TIP60 antibody. Result showed the presence of RFP-TIP60 protein only in the samples expressing GFP-PXR & RFP-TIP60 however, no signal was detected in the samples expressing GFP-PXR with RFP plasmid (Fig. 1B). To investigate endogenous PXR-TIP60 interaction, IP was performed with HepG2 cell lysate using PXR antibody or mouse IgG as a control. Immunoblotting result showed the presence of TIP60 protein only in the PXR immunoprecipitated lane while no signal was observed in control IgG lane (Fig. 1C). The ability of PXR protein to pull-down TIP60 indicated the existence of interaction between them inside the cell. In order to examine whether direct physical interaction exists between TIP60 and PXR, we performed *in vitro* pull-down assay. Expression and purification of GST-tagged PXR and His-tagged TIP60 proteins were confirmed from BL21 (DE3) *E*. *coli* strain (Supplementary Figure S2). Subsequently, BL21 (DE3) cells were cotransformed with His-TIP60 plasmid in combination with plasmid expressing GST-PXR or GST protein. Proteins were coprecipitated using glutathione sepharose beads and the precipitates were probed with anti-His antibody to detect the presence of His-tagged TIP60. Result showed that His-TIP60 was specifically pulled down by GST-PXR protein and not with GST protein under similar conditions (Fig. 1D). Together, these results demonstrate that TIP60 and PXR physically interact with each other to form a complex inside the nucleus.

### PXR interact with TIP60 through 174–210 amino acid region of its ligand binding domain

**Figure 2:**
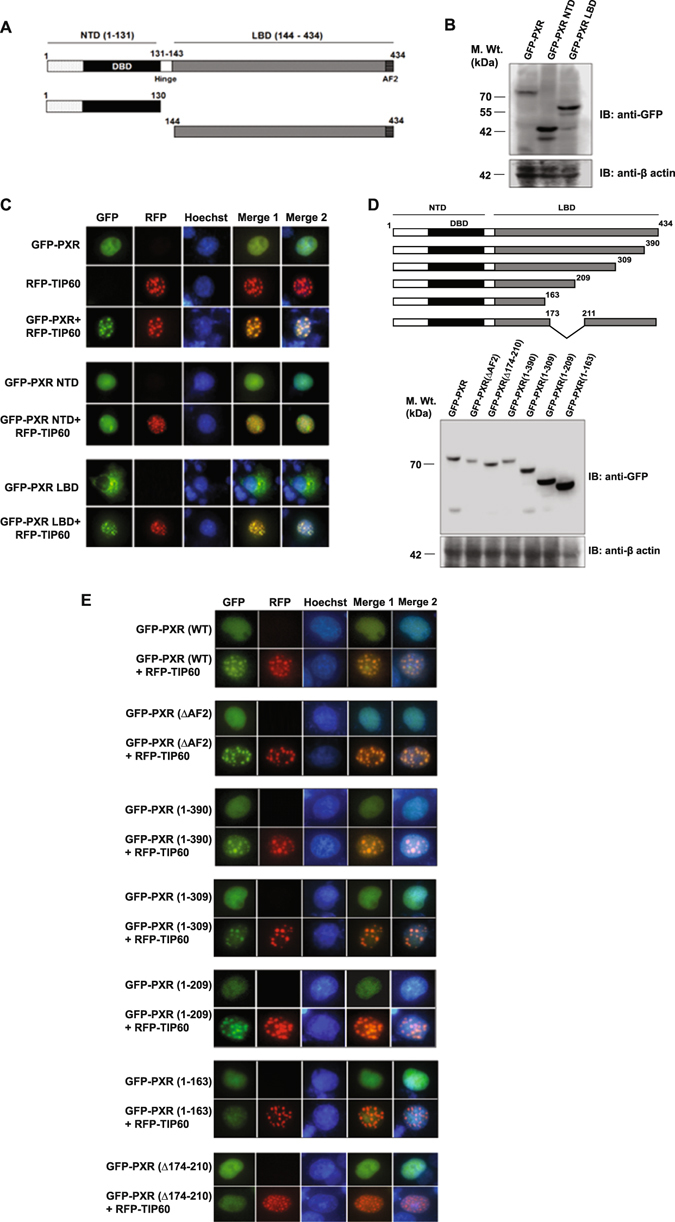
174–210 region of PXR LBD is required for its interaction with TIP60. (**A**) Schematic representation of full-length PXR and its domain-wise deletion constructs. (**B**) Western blot showing expression of full-length PXR and its deletion constructs cloned into GFP vector. (**C**) Cos-1 cells were transfected with GFP-PXR or its deletion constructs alone and in combination with RFP-TIP60 as indicated in the figure and their intracellular localization was monitored by fluorescence imaging. (**D**) Schematic representation of a series of full-length PXR C-terminal deletion constructs. Western blot analysis was performed using anti-GFP antibody to detect expression of PXR LBD deletion constructs. (**E**) GFP-PXR or its deletion constructs as indicated were transfected alone or with RFP-TIP60 in Cos-1 cells and subcellular localization of tagged proteins were monitored by live cell imaging. Full-length immunoblots are presented in Supplementary Figure S6D–E.

As our data show that TIP60 and PXR directly interact with each other we wanted to identify the region within PXR responsible for this interaction. Like other NRs, PXR consists of distinct functional domains therefore we generated domain-wise deletion mutants of PXR and cloned them into GFP vector (Fig. 2A). Expressions of all the deletion constructs of PXR were confirmed by Western blotting (Fig. 2B). In order to test the ability of these deletion constructs to form foci with TIP60, GFP-PXR (full-length), GFP-PXR NTD or GFP-PXR LBD plasmids were transfected alone or in combination with RFP-TIP60 plasmid in Cos-1 cells and imaging was performed to monitor their intracellular localization. Result revealed that only GFP-PXR LBD colocalized with RFP-TIP60 in the nucleus and formed punctate foci with TIP60 in a similar manner to GFP-PXR (full-length) (Fig. 2C). However, GFP-PXR NTD did not show any colocalization with RFP-TIP60 and remained homogenously distributed inside the nucleus (Fig. 2C).

To further determine the precise region of PXR LBD that mediates its interaction with TIP60, a series of C-terminal deletion mutants of full-length PXR protein were generated based on its secondary structure and cloned into GFP vector (Fig. 2D). Expressions of all the constructs were confirmed by Western blotting using anti-GFP antibody (Fig. 2D). Since PXR interacts with different coactivators and corepressors through its AF2 domain, we also generated AF2 deletion construct of PXR. Live cell imaging showed that GFP-PXR (ΔAF2) construct when coexpressed with RFP-TIP60 colocalized with TIP60 inside the nucleus and formed distinct nuclear foci similar to wild-type PXR protein (Fig. 2E). Subsequently, live cell imaging was performed for other C-terminal deletion constructs of PXR to test their ability to interact with TIP60. All the deletion constructs of PXR except deletion (1–163) were able to colocalize with TIP60 to form nuclear foci (Fig. 2E). Failure of PXR deletion construct (1–163) to form foci with TIP60 indicated that amino acids 164-210 might contain the region essential for interaction with TIP60. Since PXR has a natural isoform which lacks amino acids from 174–210 regions, a deletion construct lacking 174–210 amino acids from full-length PXR protein was also generated in GFP vector. Colocalization study by live cell imaging revealed that GFP-PXR (Δ 174–210) failed to colocalize with RFP-TIP60 (Fig. 2E). Together, these results show that 174–210 region of PXR LBD is important for its interaction and foci formation with TIP60.

### TIP60 interact with PXR through its NR Box and its catalytic activity is essential for TIP60-PXR foci formation

**Figure 3:**
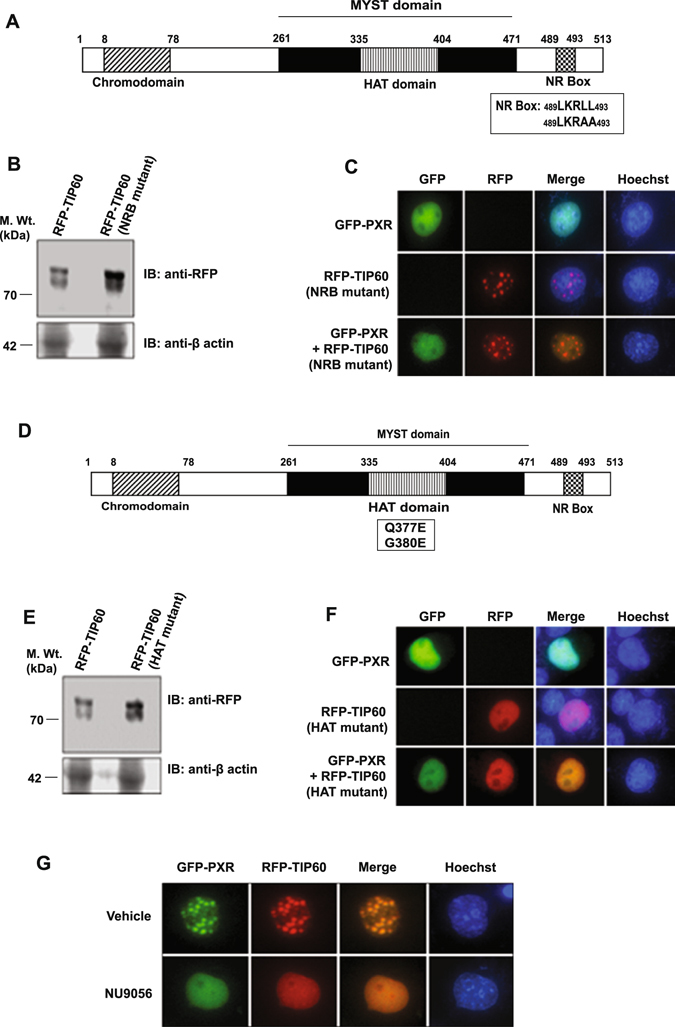
TIP60 interacts with PXR through its NR Box and its catalytic activity is required for TIP60-PXR foci formation. (**A**) Schematic figure representing domains of TIP60. The position of TIP60 NR Box (NRB) is indicated in the figure. (**B**) Western blot showing the expression of RFP-TIP60 and RFP-TIP60 (NRB mutant). (**C**) Live cell imaging was done to examine the effect of NR Box mutation in TIP60 on intracellular dynamics of PXR. Cells were transfected with RFP-tagged TIP60 NRB mutant alone or in combination with GFP-PXR and intracellular localization of the transfected proteins were monitored. (**D**) Schematic representation of different TIP60 domains and generated mutation sites in the HAT domain. (**E**) Western blot showing the expression of RFP-TIP60 and RFP-TIP60 (HAT mutant). (**F**) Live cell imaging performed with Cos-1 cells transfected with GFP-PXR and RFP-TIP60 (HAT mutant) alone or together. (**G**) Inhibition of TIP60 HAT activity by NU9056 disrupts TIP60-PXR foci. GFP-PXR and RFP-TIP60 plasmids were cotransfected in Cos-1 cells and NU9056 (2.5 µM) or vehicle (DMSO) was added to the transfected cells and live cell imaging was performed to detect their intracellular localization. Full-length immunoblots are presented in Supplementary Figure S6F.

TIP60 is known to interact with class I NRs through its NR Box, so we were interested to know whether TIP60 utilizes the same region for its interaction with PXR. NRB mutant of TIP60 was generated by converting conserved leucine residues of NR Box into alanine (LKR**LL** to LKR**AA**) and cloned into RFP vector (Fig. 3A) and expression of the generated RFP-TIP60 (NRB mutant) was confirmed by Western blotting (Fig. 3B). RFP-TIP60 (NRB mutant) was cotransfected with GFP-PXR in Cos-1 cells and was tested for its ability to associate with PXR to alter its localization. Result showed that although TIP60 NRB mutant formed nuclear punctate foci similar to the wild-type TIP60 protein, it failed to induce GFP-PXR intranuclear foci formation (Fig. 3C). This shows that NR Box of TIP60 is essential for TIP60-PXR colocalization and foci formation.

TIP60 contains a catalytic HAT domain (Fig. 3D). To identify whether the catalytic activity of TIP60 was important for TIP60-PXR foci formation, catalytically-dead mutant of TIP60 was generated by converting Q377E/G380E in its HAT domain through site-directed mutagenesis and the expression was confirmed by Western blotting (Fig. 3E). RFP-TIP60 (HAT mutant) was transfected alone or with GFP-PXR and live cell imaging was performed. Interestingly, in comparison to wild-type TIP60, HAT mutant of TIP60 did not form nuclear foci and remained homogenously distributed inside the nucleus and when coexpressed with GFP-PXR, both the proteins remained homogenously distributed inside the nucleus (Fig. 3F). To further validate the role of TIP60 HAT activity for PXR-TIP60 interaction and colocalization, NU9056 (a known inhibitor of TIP60’s HAT activity) was used to inhibit TIP60 HAT activity. Cos-1 cells cotransfected with RFP-TIP60 and GFP-PXR plasmids were treated with either vehicle (DMSO) or NU9056 and live cell imaging was performed. We observed that in presence of NU9056, TIP60 and PXR could not form nuclear foci and remained homogeneously distributed inside the nucleus (Fig. 3G). Together these results show that TIP60 HAT activity is required for TIP60 induced PXR foci formation in the nucleus.

### TIP60 acetylates PXR at lysine 170

**Figure 4:**
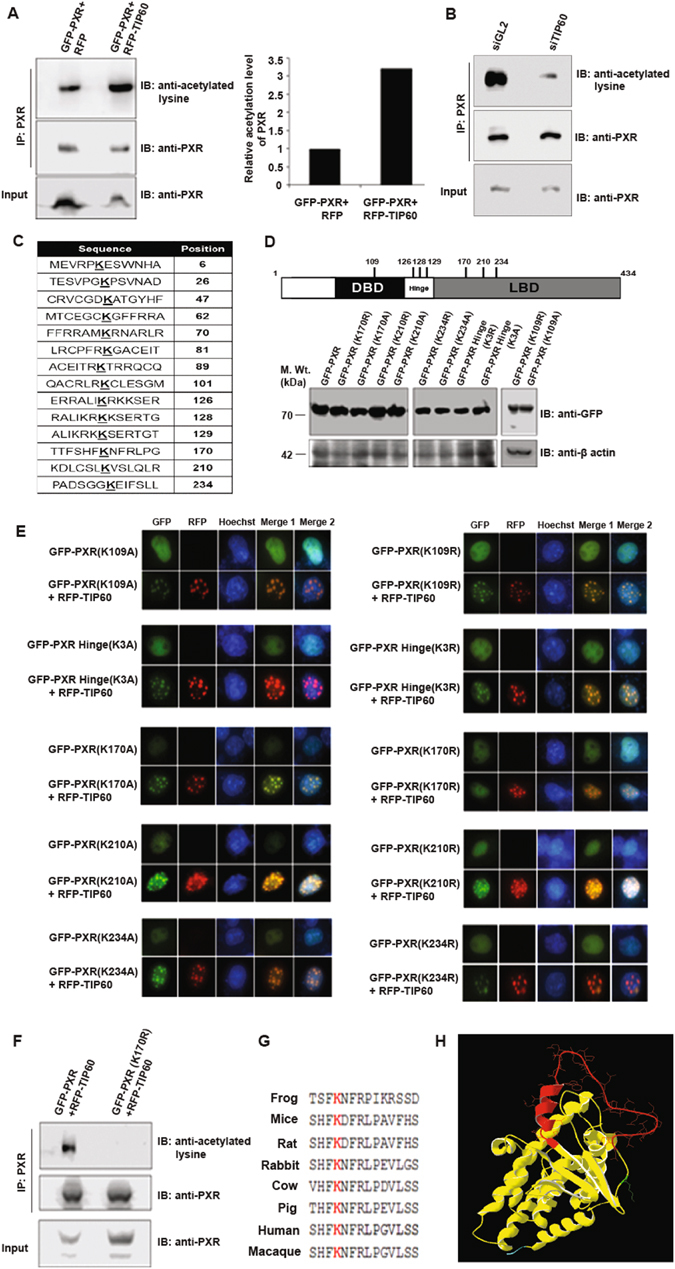
TIP60-mediated acetylation of lysine 170 of PXR is essential for TIP60-PXR foci formation. (**A**) GFP-PXR was cotransfected with RFP-TIP60 or RFP vector in Cos-1 cells. Harvested cells were subjected to immunoprecipitation with anti-PXR antibodies followed by immunoblotting performed with anti-acetylated lysine or anti-PXR antibody. Band intensities were measured using C-digit scanner and graph was plotted for obtained values. (**B**) Cells were transfected with siTIP60 or siGL2 followed by GFP-PXR transfection. Anti-PXR antibody was used to immunoprecipitate PXR followed by immunoblotting with anti-acetylated lysine or anti-PXR antibody. (**C**) Table shows the putative acetylation sites in PXR protein predicted by PAIL software. (**D**) Schematic diagram shows the putative acetylation sites in PXR selected for the study. Western blot performed with anti-GFP antibody to detect expression of PXR and its acetylation-mimicking and acetylation-deficient mutant constructs cloned into GFP vector. (**E**) Acetylation of lysine residue 170 in PXR is essential for TIP60-PXR foci formation. Cos-1 cells were transfected with GFP-PXR or its lysine mutants alone or with TIP60 as indicated followed by live cells imaging. (**F**) TIP60 acetylates lysine 170 of PXR. GFP-PXR or GFP-PXR (K170R) along with RFP-TIP60 plasmids were transfected in Cos-1 cells. Immunoprecipitation was performed similar to panel A of Fig. 4 using anti-PXR antibody. Western blotting was performed with immunoprecipitated extract using anti-PXR or anti-acetylated lysine antibodies as indicated. (**G**) Sequence alignment showed lysine 170 to be conserved in PXR ortholog across different species as depicted in the figure. (**H**) Structure of PXR LBD. Red color highlights 174–210 region while green color shows the position of lysine 170. Blue color depicts the incomplete hinge region. Full-length immunoblots are presented in Supplementary Figure S6G–J.

To investigate whether PXR can be acetylated by TIP60, GFP-PXR was cotransfected with RFP-TIP60 or RFP alone in Cos-1 cells. PXR was first immunoprecipitated with anti-PXR antibody and the immunoprecipitates were then immunoblotted with anti-PXR or anti-acetylated lysine antibody. Blot probed with anti-PXR antibody confirmed that PXR was immunoprecipitated from both the samples (Fig. 4A). Immunoblotting with anti-acetylated lysine antibody showed hyperacetylation of PXR in presence of TIP60 (Fig. 4A). Graph for the obtained band intensities showed that PXR was more than 3-fold acetylated in presence of TIP60 compared to the basal level (Fig. 4A). To further validate the effect of TIP60 on acetylation level of PXR we performed knockdown experiments using double-stranded RNA oligo’s of siTIP60 or siGL2 (as control) followed by cotransfection of GFP-PXR. PXR was immunoprecipitated using anti-PXR antibody and the blot was subsequently probed with anti-acetylated lysine antibody or anti-PXR antibody. Immunoblotting result showed drastic reduction in the acetylation level of PXR in absence of TIP60 (Fig. 4B). The efficiency of TIP60 siRNA used in the experiment was confirmed by Western blot analysis (Supplementary Figure S3).

After corroborating that PXR get acetylated by TIP60, we wanted to identify the lysine residue of PXR that gets acetylated by TIP60. PAIL software was used to locate the putative acetylation sites present on PXR protein (Fig. 4C). Since previous results demonstrated that LBD region of PXR is sufficient to interact and make nuclear foci with TIP60, we targeted the lysine residues identified within LBD and in nearby hinge region. In addition, lysine 109 present in NTD region was selected based on a recently published report^14^. All the selected lysine residues were converted either into arginine (to generate acetylation-null mutant) or into alanine (to mimic constitutively acetylated form) respectively. Expressions of all the mutants were confirmed by Western blot analysis (Fig. 4D). GFP-tagged PXR wild-type and its lysine mutants were transfected in Cos-1 cells alone or with RFP-TIP60. Live cell imaging result showed homogenous nuclear distribution of all the PXR mutants similar to its wild-type protein when expressed alone and formed colocalized foci with RFP-TIP60 except the acetylation deficient GFP-PXR (K170R) mutant which failed to do so (Fig. 4E). To validate whether lysine 170 of PXR gets acetylated by TIP60, Cos-1 cells were transfected with GFP-PXR or GFP-PXR (K170R) along with RFP-TIP60. Immunoprecipitation was performed using anti-PXR antibody from both the samples and immunoblot analysis was performed using anti-PXR or anti-acetylated lysine antibody. Result showed that PXR (wild-type) gets acetylated in presence of TIP60 while lysine 170 arginine mutant of PXR (K170R) did not show any acetylation even in presence of TIP60 (Fig. 4F).

Lysine 170 was found to be conserved in PXR ortholog from different species based on *in silico* analysis (Fig. 4G). Multiple sequence alignment showed that lysine 170 is highly specific to PXR when compared with 8 other members of NR 1I subfamily (Supplementary Figure S4). PXR LBD structural modelling showed that 174–210 region of PXR is part of a loop structure located on outer core of PXR and lysine 170 residue is also positioned within this loop (Fig. 4H).

Together, these results specify that TIP60 acetylates PXR at lysine 170 residue and TIP60-dependent acetylation of PXR is required for its intranuclear reorganization.

### RXR does not form foci with TIP60 and is not required for TIP60-PXR complex formation

**Figure 5:**
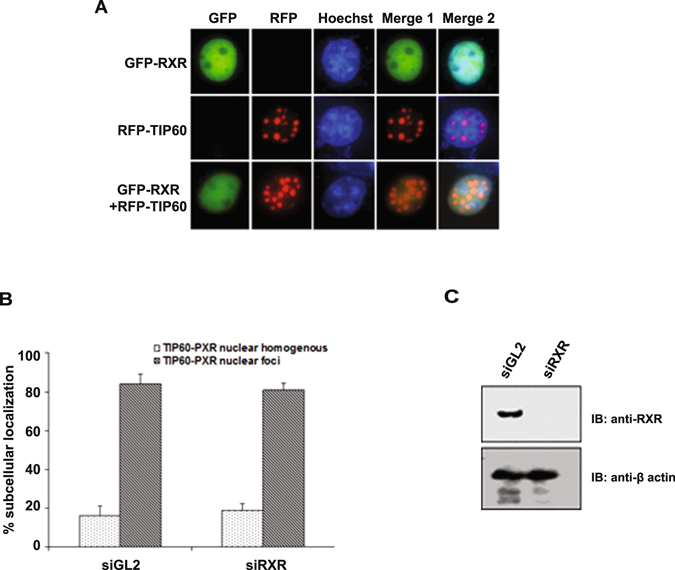
RXR does not form foci with TIP60. (**A**) Cos-1 cells were transfected with GFP-RXRα or RFP-TIP60 alone or together and live cell imaging was performed to monitor their intracellular localization. (**B**) RXR knockdown does not affect TIP60-PXR complex formation. Cos-1 cells were transfected with siGL2 (control) or siRXRα followed by RFP-TIP60 and GFP-PXR plasmids transfection. Live cell imaging was performed and at least 100 transfected cells were counted to detect TIP60-PXR foci formation. Graph depict the mean value of three different sets of experiments with ± SD. (**C**) Western blot show the knockdown efficiency of siRNAs used in the experiment. Full-length immunoblot are presented in Supplementary Figure S6K.

Since PXR exerts its transcriptional function by associating with RXR, we were interested to examine whether RXR can also associate with TIP60. To determine this possibility, Cos-1 cells were transfected with RFP-TIP60 and GFP-RXR plasmids and live cell imaging was performed. Result showed that GFP-RXR did not colocalize with TIP60 and remained homogeneously distributed inside the nucleus (Fig. 5A). Furthermore, RXR knockdown performed in Cos-1 cells using double stranded RNA oligo’s of siGL2 or siRXRα followed by cotransfection of GFP-PXR and RFP-TIP60 plasmids showed no significant difference in cell numbers forming TIP60-PXR foci under both the condition (Fig. 5B). Western blot confirmed the knockdown efficiency of RXRα siRNA used in the experiment (Fig. 5C). This suggest that RXR also a class II NR and an obligatory partner of PXR shows no colocalization with TIP60 and also does not influence the formation of TIP60-PXR complex.

### TIP60-PXR complex does not activate PXR target gene and is sensitive to PXR ligand

**Figure 6:**
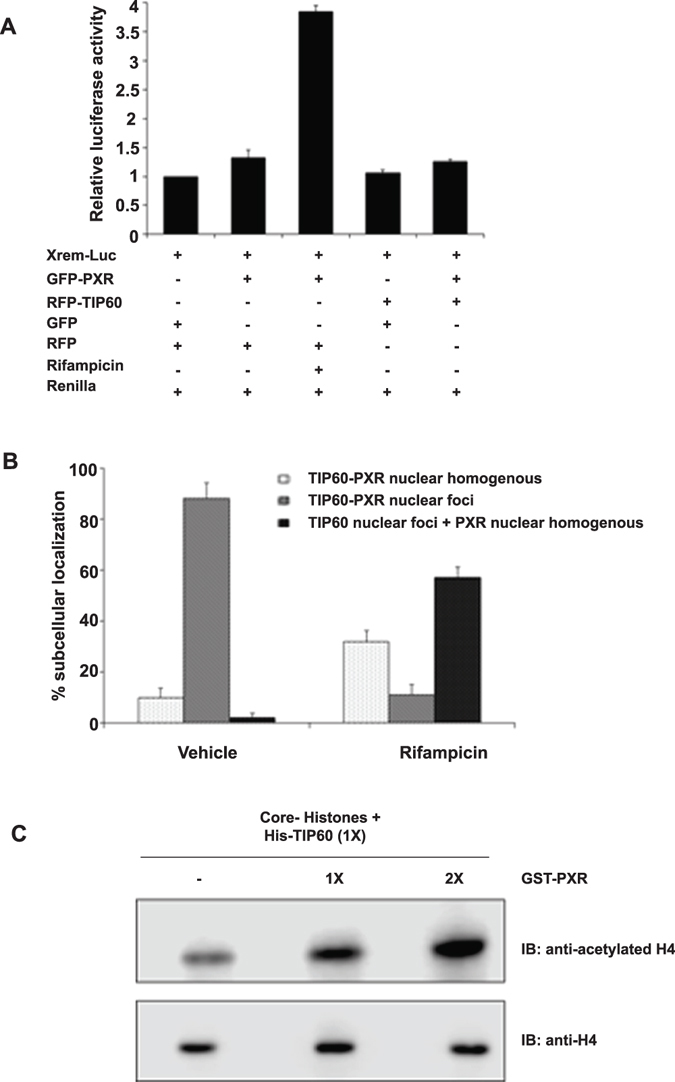
TIP60-PXR complex does not stimulate PXR-mediated transactivation. (**A**) Cos-1 cells were transfected with RFP-TIP60, GFP-PXR and XREM-Luc (containing luciferase gene under PXR targeted gene CYP3A4 promoter) plasmids and empty vectors as indicated in the figure. Rifampicin (10 µM) was added as PXR agonist. Dual-Glo luciferase assay was performed with the transfected cell lysates. Firefly luciferase values were normalized to the corresponding Renilla luciferase signal for transfection efficiency. The graph represents the normalized mean values (±SD) of three independent set of experiments performed in triplicate. (**B**) TIP60-PXR complex is sensitive to PXR agonist. To observe the effect of PXR ligand on TIP60-PXR foci formation, Cos-1 cells cotransfected with RFP-TIP60 and GFP-PXR were treated with vehicle (DMSO) or rifampicin (10 µM) and subcellular localization was monitored and recorded by fluorescence microscope. At least 100 transfected cells were counted and graph was plotted for the mean value of three independent experiments with ± SD. (**C**) TIP60 HAT activity is augmented in presence of PXR. Purified recombinant His-TIP60 and GST-PXR proteins were used for *in vitro* HAT assay using core histones as substrate. Proteins were resolved by 15% SDS-PAGE followed by immunoblotting with indicated antibodies. Full-length immunoblots are presented in Supplementary Figure S6L.

PXR is known to activate several genes involved in xenobiotic metabolism upon activation by its agonists. To examine whether TIP60-PXR complex leads to activation of PXR targeted genes luciferase assay was performed. HepG2 cells were transfected with XREM-Luc plasmid alone or in combination with GFP-PXR or RFP-TIP60 or both as indicated in the Fig. 6A. Rifampicin was used as a PXR agonist. Result showed that TIP60-PXR complex failed to induce Cyp3A4 transcription (Fig. 6A). However, under similar conditions in positive control lane, rifampicin alone activated PXR transcription by ~4 fold (Fig. 6A). Western blotting of transfected samples by indicated antibody showed the expression of proteins in the samples (Supplementary Figure S5A). To investigate the effect of PXR ligand on TIP60-PXR complex formation, Cos-1 cells were transfected with GFP-PXR and RFP-TIP60 plasmids and were treated with vehicle or rifampicin. Cell imaging was performed to count the number of cells forming TIP60-PXR foci and graph was plotted for the obtained values (Fig. 6B). Result showed that in presence of rifampicin, TIP60-PXR foci gets disrupted and PXR showed diffused distribution pattern in the nucleus. Together these results show that TIP60-PXR complex does not activate PXR targeted gene and the complex is sensitive to PXR agonist.

### PXR stimulates TIP60 catalytic activity

TIP60 has a conserved catalytic domain that facilitates transfer of acetyl group onto the lysine residues of its substrate. In an attempt to examine whether TIP60-PXR complex formation had any effect on the catalytic activity of TIP60 we performed *in vitro* HAT assays using purified recombinant His-TIP60 protein alone or with GST-PXR protein taking core histones as substrate. We observed that TIP60-mediated acetylation of histones was enhanced in presence of PXR (Fig. 6C) while under similar conditions GST-PXR or GST had no obvious effect (data not shown). This shows that formation of TIP60-PXR complex augments the TIP60 acetylation activity for histones.

### TIP60-PXR complex promotes cell migration and adhesion

**Figure 7:**
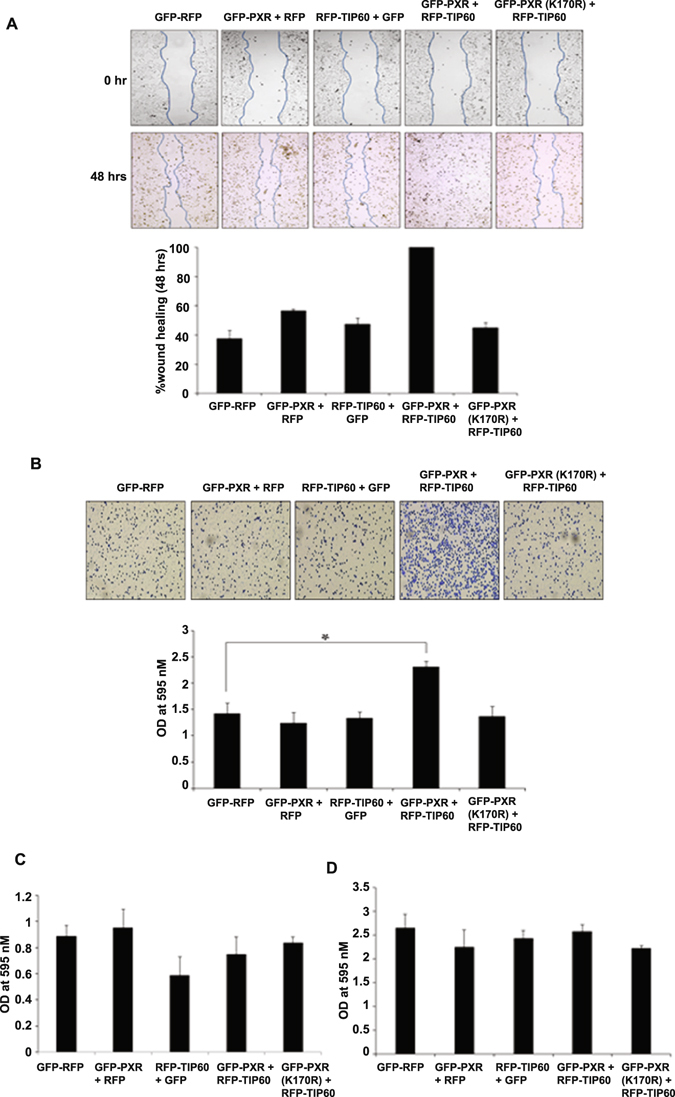
TIP60-PXR complex promotes cell migration and adhesion. (**A**) Wound-healing assay was performed in HepG2 cells transfected with different plasmids in different combinations as indicated in the figure. Scratch was generated with a sterile pipette tip in the transfected cells monolayer and the wound gap closure was monitored by capturing images at different time intervals and the graph was plotted for the obtained values of wound healing at 48 hrs. (**B**) To check the effect of TIP60-PXR complex on cell adhesion, HepG2 cells were transfected with indicated plasmids and equal numbers of cells were seeded for adherence. Adhered cells were stained with crystal violet and images were taken followed by cell lysis with SDS and the absorbance was measured at 595 nM. Graph was plotted for the average value of three independent experiments with ±SD. Asterisk shows P value < 0.005. (**C**) For examining the effect of TIP60-PXR complex on cell invasion, HepG2 cells were transfected with indicated plasmids and equal numbers of cells were seeded on geltrex coated transwell filled with 10% FBS medium in the bottom chamber. After 12 hrs cells migrated on the lower surface of the membrane were stained using crystal violet and lysed with SDS. Absorbance was measured at 595 nM and graph was plotted for the average value of three independent experiments done in duplicate with ±SD. (**D**) For cell proliferation assay, HepG2 cells were transfected with plasmids as indicated and equal number of cells were seeded. After 6–7 days cells were stained with crystal violet and lysed with SDS followed by absorbance measurement at 595 nM. Average value of three independent experiments was used to plot the graph with ±SD.

Both PXR and TIP60 have been implicated separately in driving important cellular processes like migration, adhesion, invasion and proliferation which are mostly studied in context of cancer growth and progression. Considering the growing importance of these proteins with respect to aforesaid functions we wanted to examine whether TIP60-PXR complex formation has any influence on these cellular processes. To determine the effect of TIP60-PXR complex on cell migration we performed wound healing assay using RFP-TIP60 plasmid cotransfected either with GFP-PXR or its lysine 170 mutant (K170R) in HepG2 cells. After cells became confluent a scratch was generated by a 10 µl pipette tip and the resulting gap formed between cell boundaries was monitored at different time points. We observed a faster accumulation of cells in the scratch area cotransfected with TIP60 & PXR compared to the cells expressing TIP60 or PXR alone. Cells transfected with TIP60-PXR filled the gap completely by ~48 hrs of wound generation (Fig. 7A). Under similar conditions PXR or TIP60 or lysine 170 mutant of PXR along with TIP60 did not show any significant difference in the cell migration compared to control cells transfected with GFP and RFP vectors (Fig. 7A). For measuring the effect of TIP60-PXR complex on cell adhesion, HepG2 cells were transfected with GFP-PXR alone or with RFP-TIP60 plasmid or RFP-TIP60 alone or with GFP-PXR (K170R) plasmids. Results showed that TIP60-PXR complex enhanced the adhesion property of cell compared to TIP60 or PXR alone (Fig. 7B). Similarly, effect of TIP60-PXR complex on invasive capacity of cells was evaluated. HepG2 cells were transfected with RFP-TIP60, GFP-PXR alone or in combination as indicated in Fig. 7C. In a different set, RFP-TIP60 was also transfected with GFP-PXR K170R mutant. No significant difference was observed in invasion capacity of cells either transfected with TIP60 & PXR together or TIP60 or PXR alone as compared to control cells (Fig. 7C). Similarly, we observed TIP60-PXR complex had no effect on HepG2 cell proliferation as shown in Fig. 7D. Western blot was performed to detect the expression of tagged proteins by respective antibodies for each experiment (Supplementary Figure S5B–E). Together, these results show that TIP60-PXR complex promotes migration and adhesion properties of the cell.

## Discussion

Based on previous studies we are very much aware of the role of TIP60 as a nuclear receptor coregulator that interacts specifically with class I NRs including AR, ER α, ER β & PR, class II NR PPARγ and an orphan nuclear receptor Rev-erbβ to modulate their transactivation function. Here in this report we have shown for the first time that TIP60 interacts with PXR, a prominent member of class II NR in unliganded condition and together this complex promotes cell migration & adhesion. This is an important finding in contrast to the existing view of TIP60 solely acting as a coregulator of interacting NR for their ligand-dependent functions.

PXR is a ligand-modulated transcription factor that is primarily known to regulate the genes involved in xenobiotic metabolism. However, expression of PXR in various tissues other than liver and intestine, which are not directly involved in xenobiotic metabolism in the body and an incomplete overlap between PXR and its targeted xenobiotic gene expression indicates about its alternative undiscovered functions. In absence of an accurate explanation for its tissue-specific presence and the resulting physiological or pathophysiological outcome, our understanding of PXR has remained largely limited to its role as a xenobiotic & endobiotics sensor. Deregulated PXR activity has been associated with development and progression of many metabolic disorders such as diabetes, obesity, inflammatory diseases and various cancers. Unexplained activity of PXR in different physiological and pathophysiological conditions especially in absence of any known ligand suggests that PXR may utilize alternative or combinatorial mechanisms to exert its cellular functions. In general, the activity of PXR is mediated through its interaction with different ligands however in recent time it has been recognized that PXR activity can be differentially modulated by PTMs, cross-talk with cell signaling factors and protein-protein interactions propagating the idea of ligand-independent mode of PXR activation. However, thorough investigations are required to identify the signals and characterize the molecular mechanisms operating upon PXR that may regulate its activity under unliganded condition.

Our study adds an important layer to PXR regulation by ascertaining its interaction with an important regulatory protein TIP60. We have identified that TIP60, a lysine acetyltransferase protein interacts with unliganded PXR and modulates its intracellular dynamics. It is very well known that PXR in unliganded condition remains homogenously distributed inside the nucleus however when coexpressed with TIP60, an entirely altered distribution profile is observed. Unliganded PXR forms punctate nuclear foci that perfectly colocalizes with TIP60 nuclear foci (Fig. 1A). TIP60-mediated intranuclear reorganization of PXR is found to be a result of its interaction and catalytic activity of TIP60. Domain mapping analysis revealed that the interaction of PXR with TIP60 is specifically mediated by a minimal essential region comprised of 37 amino acids (174–210) located within its LBD. Deletion of this region in PXR preclude its intranuclear reorganization and colocalization with TIP60 suggesting the importance of this region for PXR interaction with TIP60. Further, structural data analysis showed that 174–210 region makes a loop like structure which is exposed on outer core of PXR and also encompass lysine 170 residue which is identified as a target residue of TIP60. Consecutively, not only binding of this 37 amino acid region but also acetylation of lysine 170 residue by TIP60 is also equally important for TIP60-PXR foci formation. Interestingly, sequence analysis showed that this region is either absent or is found to be incomplete in all other examined class II NRs (Supplementary Figure S3). Also lysine 170 residue is not conserved in these class II receptors (Supplementary Figure S3). This shows that interaction of TIP60 with PXR is highly specific and may have some important physiological implications as many other important class II NR like TR, VDR, CAR & RXR are shown not to interact with TIP60^26, 33^. Interestingly, PXR.2 a naturally occurring splice variant and also the most abundant alternative isoform of PXR, exists that differ from the full-length PXR by not having this 37 amino acid region (174–210) in its LBD^34^. Even though PXR.2 can interact with RXR and binds to PXR response elements, it fails to show target gene transactivation for the tested ligands in human. The dissimilarity observed in the transcriptional behavior of alternative PXR isoforms could be due to the difference in their coregulators interaction profile, intracellular localization and PTMs that may have a dramatic impact on their signaling capacity. By utilizing specific isoform of PXR, cell might make a fine balance between PXR-dependent functions under different conditions.

Our study takes us closer towards understanding the specificity of this interaction taking place between TIP60 and PXR. TIP60 contains a single NR Box that mediates its interaction with different members of class I NRs and with class II NR PXR except in case of PPARγ^31^. Conversely different NRs may utilize different domains to interact with TIP60 NR Box. For instance, interaction of AR with TIP60 is mediated through its LBD in a ligand-independent manner while in case of ERα interaction ensues specifically in presence of estrogen. On contrary, ERβ can interact with TIP60 both in liganded & unliganded condition and utilizes its hinge region to interact with TIP60^28–30^. Although TIP60 employs similar NR Box for its interactions with different NRs, utilization of unique domains by these NRs for interaction with TIP60 indicates target specificity for the formed complexes on chromatin which can regulate expression of selective set of genes in each case. Recent studies have shown that p53 also interacts with PXR through the same region^35, 36^ involved in interaction with TIP60 and therefore can compete with TIP60 for interacting with PXR. TIP60 is also known to interact and modulate transcriptional activity of p53 during DNA damage. Also, in presence of tested PXR agonist the interaction of TIP60-PXR is disrupted (Fig. 6B). These findings suggest a possible crosstalk or dynamic interplay between these important regulatory proteins which might depend on the cellular microenvironment.

To gain insights into the physiological consequence of TIP60-PXR complex formation, we studied their effect on xenobiotic metabolism function of PXR and observed that TIP60-PXR complex does not activate gene normally known to be target of ligand-activated PXR (Fig. 6A). This was not surprising, as the TIP60-PXR complex is formed only in absence of PXR agonist and presence of PXR ligand rifampicin disrupted this interaction. Other studies have also shown that PXR agonist significantly decreases PXR acetylation which corresponds to our findings^12–14^. Also, TIP60-mediated intranuclear foci formation of unliganded PXR is remarkably different from the distribution profile of PXR in the apo- or agonist-bound state, indicating the involvement of this novel complex in unidentified functions. Being a chromatin modifier protein TIP60 serves as transcriptional coregulator for many genes. TIP60 is known to acetylate histone tails and histone acetylation is often associated with decompaction of chromatin making it accessible to transcription factors to promote transcriptional activation. Intriguingly, our HAT assay result shows that PXR augments the catalytic activity of TIP60 for histone substrates and leads to hyperacetylation of histones. This suggests that TIP60-PXR complex binds to promoter of the target genes and may regulate the chromatin microenvironment by modulating acetylation state of histones and thus favoring active transcription of targeted genes. Based on these results we speculate that in contrast to PXR-RXR complex that activates xenobiotic pathway-related genes, TIP60-PXR complex might bind to promoters of totally different set of genes to modulate their transcription. It would be fascinating to investigate where does this complex gets recruited on genome and what set of genes and signaling pathways are regulated by this complex.

Since TIP60-PXR formed colocalized nuclear foci in several dissimilar cell lines under normal growth conditions tested in the study we anticipate that it might be a ubiquitous phenomenon to cells. Both TIP60 and PXR are independently shown to have implications in proliferation, migration and invasion which are important processes for normal cell activities as well as in certain pathophysiological conditions. We observed that TIP60-PXR complex enhanced migratory and adhesion properties of the cell (Fig. 7A,B). In multicellular organisms cell migration and adhesion are important fundamental processes required starting from establishment & development to maintaining proper cellular organization in embryo and in adult for tissue repair, mounting proper immune response and in maintaining tissue homeostasis. Also in cancer aberrant cell migration occurs during metastasis^37–39^. Genome wide siRNA screen in *Caenorhabditis elegans* have identified TIP60 as one of the ‘hub’ gene out of total six identified hubs that controls multiple signaling pathways showing indispensable and multifunctional role of TIP60^40^. In this regard, ability of TIP60-PXR complex to regulate migration & adhesion ability of cell may have substantial importance and hence require thorough investigation to identify what set of genes and signaling pathways are regulated by this complex to understand what physiological or pathophysiological consequences would result on manipulating their association.

**Figure 8:**
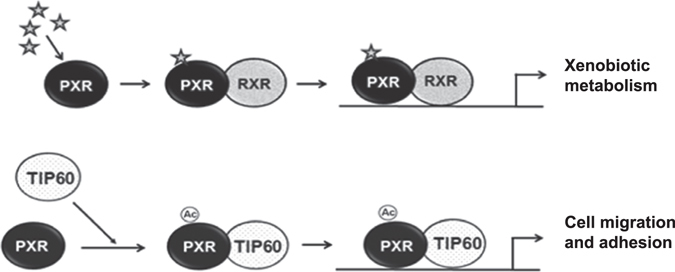
Model depicting selective interaction of unliganded PXR with TIP60. In general, PXR gets activated in presence of its ligand and forms a heterodimeric complex with its partner RXR and binds to the promoter of its target genes to induce their transactivation. This model proposes that under anonymous conditions TIP60 may induce the intranuclear reorganization of unliganded PXR and form a complex. Together, the novel complex of TIP60 & PXR promotes cell migration & adhesion via regulating the pathways that remains to be determined. Unliganded PXR in complex with TIP60 may perform alternative physiological functions within the cell other than the recognized ligand-dependent functions suggesting that the combinatorial effect of the cellular levels of these important coregulatory proteins along with cellular microenvironment may regulate their behavior and functional outcome in a context-dependent manner. Star in the figure depicts PXR ligand and (Ac) denotes acetylation mark.

Based on the present findings, we propose a model that suggests a possible mechanism by which TIP60 modulates subcellular dynamics of unliganded PXR and together this novel complex of TIP60 and PXR enhances migration and adhesion properties of cell (Fig. 8). PXR contains a minimal essential region that serves as a scaffold to anchor TIP60. Intranuclear reorganization of unliganded PXR and its interaction with TIP60 is dependent on catalytic activity of TIP60. Correspondingly, TIP60-PXR complex formation leads to enhanced HAT activity of TIP60 suggesting that this complex may induce hyperacetylation of histones on promoter chromatin leading to active transcription of genes regulating cell migration & adhesion. Apart from promoting cell migration and adhesion, together this complex may perform functions that we are still not aware of at this point.

## Methods

### Cell culture

HepG2 (human liver cell line), Cos-1 (monkey kidney cell line) and MCF-7 (human breast cancer cell line) were grown in Dulbecco’s Modified Eagle’s Medium (DMEM) supplemented with 10% fetal bovine serum (FBS) and penicillin-streptomycin. HCT116 cells (human colorectal carcinoma cell line) were maintained in McCoy’s media supplemented with 10% FBS and antibiotics. Subconfluent cultures were maintained at 37 °C in a humidified incubator with 5% CO_2_ and 95% air atmosphere. Charcoal stripped FBS (5%) containing medium was used to culture transfected cells.

### Primers, Plasmids, siRNAs and Transfection

All the primers were synthesized and purchased from IDT (Supplementary Table 1). RFP-TIP60 clone was generated by amplification of TIP60 ORF using pET28a-hTIP60 construct (a kind gift from Prof. Anindya Dutta, University of Virginia) as a template and cloned in pDsRed vector. GFP-PXR plasmid and GST-PXR plasmid were generated using pSG5-hPXR clone (a kind gift from Prof. Steve Kliewer of UT Southwestern Medical Center) as a template for amplification of the ORF and subsequent cloning in pEGFP and pGEX6p2 vector. GFP-RXRα clone was generated by using pBABE puro human RXRα clone (a gift from Ronald Kahn (Addgene plasmid # 11441)) as a template for the amplification of RXRα ORF. Mutants and deletions of PXR and TIP60 were generated by overlapping PCR method using mutation-specific primers on the backbone of wild-type ORF.

siRNA for RXRα was purchased from Santa Cruz (SC-36447) while siRNA duplex for TIP60 (target sequence-TGATCGAGTTCAGCTATGA) and GL2 (target sequence-CGTACGCGGAATACTTCGA) were synthesized from Sigma. Plasmid transfections were carried out using Lipofectamine 2000 transfection reagent (Invitrogen) as per manufacturer’s instructions. Similarly, for siRNA transfection, 30 nM of annealed siRNA duplex were transfected using Lipofectamine RNAi Max reagent (Invitrogen) following the protocol described by the manufacturer. DMSO and Rifampicin were purchased from Sigma-Aldrich while NU9056 was purchased from Tocris.

### Live cell imaging

To determine the localization of fluorescent-tagged proteins, cells were transfected with GFP and RFP- tagged constructs using Lipofectamine 2000 reagent (Invitrogen). The medium was changed 5 hrs post-transfection with DMEM containing 5% charcoal-stripped FBS and incubated for 24 hrs. Live cell imaging was performed using fluorescence microscope (Nikon Eclipse Ti Advanced microscope). Hoechst (Invitrogen) was added to facilitate the visualization of nucleus.

### Western Blot analysis

For Western blotting, transfected cells were harvested and lysed in lysis buffer (50 mM Tris-Cl (pH-8.0), 150 mM NaCl, 1 mM DTT, 5 mM EDTA, 0.5% NP-40 and 1X protease inhibitor cocktail (Amresco) followed by sonication. Protein samples were resolved by SDS-PAGE, transferred onto the methanol-charged PVDF membrane using semi-dry transfer apparatus. Subsequently, the blots were probed with indicated primary antibodies. RFP antibody, GFP antibody, GST antibody, PXR antibody, His antibody and acetylated-lysine antibody were purchased from Santa Cruz Biotechnologies. TIP60 antibody, RXRα antibody and β-actin antibody were procured from Cell Signaling Technology (CST). Acetylated Histone H4 and Histone antibodies were purchased from Millipore. Horseradish peroxidase-conjugated (HRP) anti-rabbit and anti-mouse secondary antibodies were purchased from Santa Cruz Biotechnologies while light chain specific HRP-conjugated anti-rabbit and anti-mouse secondary antibodies were purchased from Millipore. Blots were developed using ECL reagent (BioRad) and C digit scanner (Licor).

### Immunoprecipitation and *in vivo* acetylation assay

For immunoprecipitation, transiently transfected cells were harvested and lysed in the following buffer- 20 mM Tris-Cl (pH-8.0), 150 mM NaCl, 2 mM EDTA, 0.5% Triton X-100, 0.1% SDS with protease inhibitor cocktail for 30 minutes at 4 °C and subsequently sonicated. Cell lysates were incubated with anti-PXR antibody for overnight at 4 °C followed by addition of equilibrated protein A Sepharose beads (Thermo Scientific) for 2 hrs. Bound beads were washed 3 times with 1 ml lysis buffer, and bound proteins were eluted by boiling the beads in SDS-PAGE sample buffer. Western blot analyses were performed using anti-PXR and anti-TIP60 antibody. For *in vivo* acetylation assay, Cos-1 cells were transfected with GFP-PXR and RFP-TIP60 or RFP plasmids. 24 hrs after medium change cells were harvested and lysed in IP buffer followed by PXR immunoprecipitation. Western blotting was performed using anti-acetylated lysine or anti-PXR antibody.

### Protein purification and glutathione S transferase (GST) pull-down assay

For purification of His-tagged protein from bacteria, transformed *E*. *coli* BL21 DE3 codon plus cells were grown at 37 °C till the optical density (OD) of the culture reached 0.6. Isopropyl-β-D-thiogalactopyranoside (IPTG) was added to final concentration of 1 mM for induction of recombinant protein expression for 15 hrs at 16 °C. Cell pellets were lysed in ice-cold lysis buffer (1X PBS, 2 mM EDTA, 5 mM DTT, 0.5 mM PMSF, 1% Triton X-100, 10% glycerol and protease inhibitor cocktail). Lysozyme (10 mg/ml) was added to the lysate and incubated at 37 °C for 20 minutes and 3 cycles of sonication were performed at 4 °C followed by addition of Triton X-100 (0.1% concentration). Lysate was rotated at 4 °C for 30 minutes followed by centrifugation at 14,000 rpm for 30 minutes and clear lysate was separated. Equilibrated Ni-nitrilotriacetic acid (NTA) beads were added and slurry was incubated for 1 hr at 4 °C by continuous rotation. Protein-bound Ni-NTA beads were separated by centrifugation and were washed with lysis buffer containing 20 mM imidazole three times. Bound proteins were eluted by lysis buffer containing 500 mM imidazole for 30 minutes at 4 °C. Eluted proteins were dialyzed in dialysis buffer (50 mM Tris-Cl (pH-8.0), 2 mM EDTA, 100 mM NaCl, 0.2 mM PMSF) and aliquots were stored at −80 °C. GST-fused PXR protein was also purified following similar method but with few changes. Transformed bacterial culture was grown till OD reached 1.5 followed by heat shock at 47 °C for 20 minutes. Recombinant protein was induced by adding 1 mM IPTG in the culture at 16 °C for 12 hrs. Lysis was done in lysis buffer (1X PBS, 2 mM EDTA, 5 mM DTT, 100 μM PMSF) and recombinant protein was trapped using glutathione agarose beads. Bound protein was then eluted using elution buffer (50 mM Tri-Cl (pH-8.0), 10 mM glutathione, 100 µM PMSF and 10% glycerol) at 4 °C for 30 minutes.

For GST pull-down assay *E*. *coli* cells (BL21 DE3 codon plus) were cotransformed with pGEX6p2-PXR or pGEX6p2 plasmids along with pET28a-TIP60 plasmid. GST-tagged proteins were purified using glutathione agarose beads following above mentioned protocol. Protein-bound beads were washed 3 times with wash buffer (1X PBS, 0.2% Triton X-100, 300 mM NaCl) and bound proteins were eluted by boiling the beads in 2X SDS sample buffer. Immunoblotting was performed using anti-GST and anti-His antibodies.

### Luciferase assay

For luciferase assay, Cos-1 cells were seeded onto 12-well plate and transfected with GFP-PXR, RFP-TIP60 plasmids alone or together with promoter-reporter plasmid XREM-Luc. Renilla luciferase plasmid was transfected as control. Following the transfection period, cells were supplemented with 5% charcoal-stripped medium and treated with DMSO (vehicle) or Rifampicin (10 µM) and incubated further for 24 hrs. To determine the transcriptional activity luciferase assay was performed using Dual glow luciferase assay kit (Promega) as per manufacturer’s protocol using single tube luminometer machine from Berthold.

### HAT assay

HAT assay was performed as described elsewhere^41^. Purified recombinant His-TIP60 and GST-PXR proteins were dialyzed in HAT assay buffer (50 mM Tris-Cl (pH-8.0), 10% glycerol, 0.1 mM EDTA, 1 mM DTT) for 3 hrs at 4 °C. Reaction was carried out in 50 µl reaction volume containing 100 µM acetyl coenzyme A, 0.5 µg core histone and recombinant proteins. Reaction mix was incubated at 30 °C for 30 minutes and reaction was stopped by adding 2X SDS loading buffer. Proteins were resolved in 15% SDS-PAGE followed by immunoblotting using anti-acetylated histone H4 antibody or histone H4 antibody.

### Sequence alignment and homology modelling

To examine the conservation of lysine at 170 position (K170) and region 174–210 in human PXR, multiple sequence alignment for PXR sequence across varied species was performed using Clustal Omega^42^. Similar analysis was also carried out in few prominent nuclear receptor 1I subfamily members. Presently, complete structure of PXR in human is not available. To understand the structural organization of human PXR, especially with K170 and 174–210 region located within LBD, the structure was modeled using homology modeling with SWISS-MODEL program^43^. While the available crystal structure of human PXR are in complex form bound to ligands with the partial DBD & LBD regions and in them the region from 174–210 residues has a small gap from 179–185 residues. The complete LBD structure predicted shares 97.5% homology with the tertiary bound form of human PXR LBD to adnectin 1 and compound 1 (PDB ID: 4S0T). The human PXR LBD bound with adnectin 1 and compound 1 (PDB code: 4S0T) crystal structure was used as template to model the LBD region of PXR. Further superimposition of modeled LBD region of PXR with the crystal structure of the LBD of human PXR resulted in rmsd of 0.06 Å, implies that the model can be used to understand the structural organization of LBD region.

### Wound healing assay and invasion assay

For wound healing assay, HepG2 cells were cultured in 6-well plate to 80% confluency and then transfected with indicated plasmids. 24 hrs after medium change, scratch was generated by 10 µl pipette tip and gap filling of the generated wound was measured at 0, 24 and 48 hrs time point using Leica microscope. HepG2 cells were transfected with plasmids as indicated and kept for 24 hrs after medium change for invasion assay. Cells were trypsinized and 40,000 cells were seeded in ECM coated transwells with 0.5% charcoal-stripped FBS containing medium while 10% FBS containing medium was added outside of the transwell as chemoattractant. After 12 hrs cotton swab was used to remove the cells from inside the transwell chamber. Cells on outer surface of transwell were stained with crystal violet and lysed with 1% SDS and absorbance was measured by taking reading at 595 nM using microplate reader.

### Colony formation and Cell adhesion assay

HepG2 cells were transiently transfected with GFP-PXR, RFP-TIP60 alone or together. GFP and RFP expression plasmids were used as control. Cells were kept for 24 hrs after medium change and then trypsinized and 10,000 cells were seeded in 6-well plate. After 6–7 days, colonies were dissolved in 1% SDS and absorbance at 595 nM was measured using microplate reader. For cell adhesion assay, HepG2 cells were transfected with indicated plasmids. Medium was replaced with 1 ml DMEM supplemented with charcoal-stripped FBS and 0.6 million transfected cells were added on geltrex (300 µg/ml) coated 6 well plates for 30 minutes at 37 °C in 5% CO_2_ environment. After incubation plate was taken out and medium was aspirated to remove unattached cells and washed with 1X PBS. Attached cells were fixed with ice-cold methanol and then stained with 0.5% crystal violet. Subsequently, cells were lysed in 1% SDS and absorbance was measured at 595 nM by microplate reader.

## Electronic supplementary material

Supplementary information
