# Supplementary material for: Novel complex of HAT protein TIP60 and nuclear receptor PXR promotes cell migration and adhesion
Source: Sci Rep. 2017 Jun 16;7:3635. doi: 10.1038/s41598-017-03783-w (PMC5473911; doi:10.1038/s41598-017-03783-w)
Supplement: Supplementary file 1 — Supplementary information [file 41598_2017_3783_MOESM1_ESM.pdf]

Title: Novel complex of HAT protein TIP60 and nuclear receptor PXR promotes cell migration and adhesion

Authors: Karishma Bakshi[1], Ranjitha B[1], Shraddha Dubey[1], Jaisri Jagannadham[1], Bharti Jaiswal[1], Ashish Gupta[1*]

Figure legends

Supplementary Figure S1: Intracellular localization of RFP-TIP60 & GFP-PXR in various cell lines. Graphs show the percentage intracellular localization of RFP-TIP60 and GFP-PXR proteins in various cell lines as indicated. Cells were transfected with RFP-TIP60 and GFP-PXR plasmids alone or together and at least 100 transfected cells were counted in each case. Graphs represent the average values of three independent experiments with ±SD

Supplementary Figure S2: Expression and purification of recombinant proteins from bacteria. *E.coli* BL21 DE3 codon plus cells were transformed with pET28a-TIP60 or pGEX6P2-PXR plasmids. IPTG was used to induce the production of recombinant proteins followed by affinity purification method as mentioned in material and method section. Coomassie gel shows the expression and purification profile of recombinant proteins. Asterisk shows the purified protein band of recombinant proteins.

Supplementary Figure S3: Western blot for TIP60 knockdown. Cells were transfected with siTIP60 or siGL2 followed by transfection with GFP-PXR. Immunoblotting was performed using anti-TIP60 or anti-β actin antibody for the samples as indicated.

Supplementary Figure S4: Multiple sequence alignment of different members of class II NRs. Clustal omega analysis was performed to identify sequence similarity between different class II NRs protein sequence. Red highlighted residue shows the corresponding amino acids to PXR lysine 170 and yellow highlighted residue shows the corresponding amino acids to PXR 174-210 region in other class II NRs.

Supplementary Figure S5: Immunoblots showing expression of tagged proteins. (A) For promoter reporter assay cells were transfected with plasmids as indicated. Lysates of the transfected cells were resolved followed by Western blotting with the indicated antibody. (B), (C), (D) & (E) shows the expression of tagged proteins by immunoblotting by indicated antibody in scratch assay, cell adhesion assay, cell proliferation assay and cell invasion assay respectively.

Supplementary Figure S6: Full-length immunoblots. Full-length blot images with indicated antibody.

Supplementary Figure S1: Graphs depicting TIP60-mediated nuclear reorganization of unliganded PXR in various cell lines.

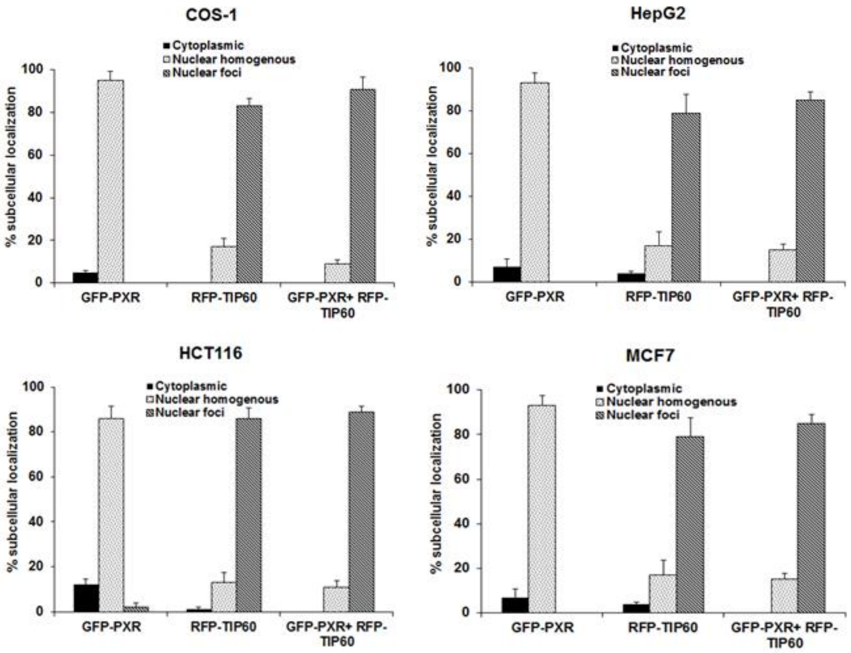

Supplementary Figure S2: Expression and purification of His-TIP60 and GST-PXR recombinant proteins from *E. coli*

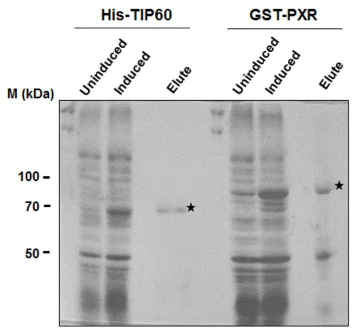

Supplementary Figure S3: Western blot of TIP60 knockdown

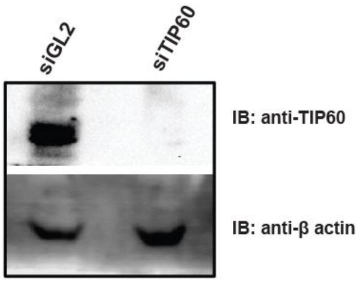

Supplementary Figure S4: Multiple sequence alignment of class II NRs

```
CAR_human        -----------------------------------MELLEEDLTCPICCSLFDDP-----
TSH_human        ------------------------------------------------------------
PPARα_human      ----MVD-----TESPLCPLSP---------------LEAGDLESPLSEEFLQE-MGNIQ
PPARγ_human      ----------------------------------------SSISTPHYED----------
RXR_human        ------------------------------------------------------------
PXR_human        ------------------------------------------------------------
VDR_human        ------------------------------------------------------------
FXR_human        MVMQFQGLENPIQISPHCSCTPSGFFMEMMSMKPAKGVLTEQVAGPLGQNLEVEPYSQYS
LXR_human        -----------------------------MS-SPTTSS----LDTPLPGNGPPQ------

CAR_human        -----RVL---PCSHNFCKKCLEGILEGSVRNSLWRPAPF-KCPTCRKETSATGINSLQV
TSH_human        ---------------------MEQ-----KP----------------------SKVEC--
PPARα_human      EISQSI---GEDSSGSFGFTEYQY-----LGSCPGSDGSVI-----------TDTLSP--
PPARγ_human      -IPFTR---TDP-----VVADYKY-----DLKLQEYQSAI--------------KVEP--
RXR_human        ------------------------------------------------------------
PXR_human        ---------------------MEV-----RPKESWNHAD----------------FVH--
VDR_human        -------------------------------------------------------MEA--
FXR_human        NVQFPQVQPQISSSSYYSNLGFYP-----QQPEEWYSPGIYELRRMPAETLYQGETEV--
LXR_human        --------PGAPS----SSPTVKE-----EGPEPWPGGPDPDVPGTDEASSACSTDWV--

CAR_human        NYSLKGIVEKYNKIKISPKMPVCKGHLG----QPLNIFCLTDMQLICGICATRGE--HTK
TSH_human        ----GSDPEENSARSPDGKRKRKNGQCSLKTSMSGYIPSYLDKDEQCVVCGDKATGYHYR
PPARα_human      ----ASSPSS------VTY-PVVPGS--------VDESPSGALNIECRICGDKASGYHYG
PPARγ_human      ----ASPPYY------SEKTQLYNKP--------HEEPSNSLMAIECRVCGDKASGFHYG
RXR_human        ------------------------------------------------------------
PXR_human        ----CEDTES------VPGKPSVNA---------D---EEVGGPQICRVCGDKATGYHFN
VDR_human        ----MAASTS------LP----DPG---------D---FDRNVPRICGVCGDRATGFHFN
FXR_human        ----AEM--P------VTKKPRMGAS--------A---GRIKGDELCVVCGDRASGYHYN
LXR_human        ----IPDPEE------EPERKRKKGP--------A---PKMLGHELCRVCGDKASGFHYN

CAR_human        HVFCSIEDAYAQERDA------FESLF-----QSFETWRRGDALSRLDT-LETSKRKSLQ
TSH_human        CITCEGCKGFFRRTIQKNLHPTYSCKY-DSCCVIDKITRNQCQLCRFKKCIAVGMAMDLV
PPARα_human      VHACEGCKGFFRRTIRLK--LVYDKC--DRSCKIQKKNRNKCQYCRFHKCLSVGMSHNAI
PPARγ_human      VHACEGCKGFFRRTIRLK--LIYDRC--DLNCRIHKKSRNKCQYCRFQKCLAVGMSHNAI
RXR_human        ------------------------------------------------------------
PXR_human        VMTCEGCKGFFRRAMKRN--ARLRCPFRKGACEITRKTRRQCQACRLRKCLESGMKKEMI
VDR_human        AMTCEGCKGFFRRSMKRK--ALFTCPFN-GDCRITKDNRRHCQACRLKRCVDIGMMKEFI
FXR_human        ALTCEGCKGFFRRSITKN--AVYKCKN-GGNCVMDMYMRRKCQECRLRKCKEMGMLAECM
LXR_human        VLSCEGCKGFFRRSVVRGGARRYACRG-GGTCQMDAFMRRKCQQCRLRKCKEAGMREQCV

CAR_human        LLTKDSDK--VKEFF-EKL--------QHTLD---------------------------Q
TSH_human        LDDSKRVA--KRKLIEQNRERRRKEEMIRSL----------------------------Q
PPARα_human      RFGRMPRS--EKAKLKAEILTC----HDIED-----------------------------
PPARγ_human      RFGRMPQA--EKEKLLAEISS-----DIDQLN----------------------------
RXR_human        ------------------------------------------------------------
PXR_human        MSDEAVEE--RRALIKRKKSERT---GTQPLGV---------------------------
VDR_human        LTDEEVQR--KREMILKRKEEEA---LKDSL-R---------------------------
FXR_human        YTGLLTEIQCKSKRLRKNVKQHADQ-TV---NE-----DSEGRDLRQ----VTSTTKSCR
LXR_human        LSEE----QIRKKKIRKQQQESQSQ-SQSPVGPQGSSSSASGPGASPGGSEAGSQGSGEG

CAR_human        KKNEILSDFETMKLAVMQAYDPEINKLNTILQEQRMAFNIAEAFKDVSEPIVFLQQMQEF
TSH_human        QRPEPTPEEWDLIHIATEA--------------HRSTNAQGSHWKQ----------RRKF
PPARα_human      ---SETADLKSLAKRIYEA--------------YLKNFNM--NKVK----------ARVI
PPARγ_human      ---PESADLRALAKHLYDS--------------YIKSFPL--TKAK----------ARAI
RXR_human        ------------------------------------------------------------
PXR_human        --QGLTEEQRMMIRELMDA--------------QMKTFDT--TFSH----------FKNF
VDR_human        --PKLSEEQQRIIAILLDA--------------HHKTYDP--TYSD----------FCQF
FXR_human        EKTELTPDQQTLLHFIMD------------------------SYNK----------QR--
LXR_human        EGVQLTAAQELMIQQLVAA--------------QLQCNKR--SFSD----------QPKV

CAR_human        REKIKVIKETPLPP----------------------------SNLPASPLMKNFDTSQWE
TSH_human        -LPDD---------------------------------------------------IGQS
PPARα_human      -----L-SGKA---------SNNPPFVIHDMETLCMA----------------EKTLVAK
PPARγ_human      -----L-TGKT---------TDKSPFVIYDMNSLMMG----------------EDKIKFK
RXR_human        ------------------------------------------------------------
```

```
PXR_human           RLPGVLSSGCELPESLQAPSREEAAKWSQVRKDLCSLKVSLQL--------RGEDGSVW-
VDR_human           RPPVRVNDGGGSHPSR--PNSRHTPSFSGDSSSSCSDHCITSSDMMDSSSFSNLDLSEE-
FXR_human           --------------------------------------------------------MPQ-
LXR_human           --------------------------------------------------------TPW-


CAR_human           DIKLVDVDKLSLPQDTGTFISKIPWSFYKLFLLILLLGLVIVFG--PTMFLEWSLFDDLA
TSH_human           PI-VSMPD--------GDKVDLEAFSEFTK-IITPAITRVVDFAKKLPMFSELPCEDQII
PPARα_human         L-VANGIQ--------NKEAEVRIFHCCQC-TSVETVTELTEFAKAIPGFANLDLNDQVT
PPARγ_human         HITPLQEQ--------SKEVAIRIFQGCQF-RSVEAVQEITEYAKSIPGFVNLDLNDQVT
RXR_human           -----------------------------------------------------PLDDQVI
PXR_human           NYKPPADS--------GGKEIFSLLPHMAD-MSTYMFKGIISFAKVISYFRDLPIEDQIS
VDR_human           DSDDPSVT--------LELSQLSMLPHLAD-LVSYSIQKVIGFAKMIPGFRDLTSEDQIV
FXR_human           EITNKILK--------EEFSAEENFLILTE-MATNHVQVLVEFTKKLPGFQTLDHEDQIA
LXR_human           PLGA-DPQ--------SRDARQQRFAHFTE-LAIISVQEIVDFAKQVPGFLQLGREDQIA
                                                                        *::

CAR_human           TWKGCLSNFSSYLTKTADFIEQSVFYWEQVTDGFFIFNERF--KNFTLVVLNNVAEFVCK
TSH_human           LLKGCCMEIMSLR-AAVRYDP-ESDTLTLS-GEMAVKREQLKNGG-LGVVSDAIFELGKS
PPARα_human         LLKYGVYEAIFAM-LSSVMN-KDGMLVAYG-NGFITREFLKSLRKPFCDIMEPKFDFAMK
PPARγ_human         LLKYGVHEIIYTM-LASLMN-KDGVLISEG-QGFMTREFLKSLRKPFGDFMEPKFEFAVK
RXR_human           LLRAGWNELLIAS-FSHRSIAVKDGI--LLATGLHVHRNSAHSAGVGAIFDRVLTELVSK
PXR_human           LLKGAAFELCQLR-FNTVFN-AETGTWECGRLSYCLE---DTAGGFQQLLLEPMLKFHYM
VDR_human           LLKSSAIEVIMLR-SNESFT-MDDMSWTCGNQDYKYRVSDVTKAGHSLELIEPLIKFQVG
FXR_human           LLKGSAVEAMFLR-SAEIFN-KKLPSG-----HSDLLEERIRNSGISDEYITPMFSFYKS
LXR_human           LLKASTIEIMLLE-TARRYN-HETECITFL-KDFTYSKDDFHRAGLQVEFINPIFEFSRA
                      :   :              .                                .:

CAR_human           YKLL--------------------------------------------------------
TSH_human           LSAFNLDDTEVALLQAVLLMSTDRSGLLCVDKIEKSQEAYLLAFEHYVNHRKHNI--PHF
PPARα_human         FNALELDDSDISLFVAAIICCGDRPGLLNVGHIEKMQEGIVHVLRLHLQSNHPDD--IFL
PPARγ_human         FNALELDDSDLAIFIAVIILSGDRPGLLNVKPIEDIQDNLLQALELQLKLNHPES--SQL
RXR_human           MRDMQMDKTELGCLRAIVLFNPDSKGLSNPAEVEALREKVYASLEAYCKHKYPEQ--PGR
PXR_human           LKKLQLHEEEYVLMQAISLFSPDRPGVLQHRVVDQLQEQFAITLKSYIECNRPQPAHRFL
VDR_human           LKKLNLHEEEHVLLMAICIVSPDRPGVQDAALIEAIQDRLSNTLQTYIRCRHPPPGSHLL
FXR_human           IGELKMTQEEYALLTAIVILSPDRQYIKDREAVEKLQEPLLDVLQKLCKIHQPEN--PQH
LXR_human           MRRLGLDDAEYALLIAINIFSADRPNVQEPGRVEALQQPYVEALLSYTRIKRPQD--QLR
                       :

CAR_human           ------------------------------------------------------------
TSH_human           WPKLLMKEREVQSSILYKGAAAE---GRPGGSLGVHPEGQQLLGMHVVQGPQVRQLEQQL
PPARα_human         FPKLLQKMADLRQLVTEHAQLVQIIKK-TESDAALHPLLQEIYRDMY-------------
PPARγ_human         FAKLLQKMTDLRQIVTEHVQLLQVIKK-TETDMSLHPLLQEIYKDLY-------------
RXR_human           FAKLLLRLPALRSIGLKCLEHLFFFKLIGD--TPIDTFLMEMLEAPH-------------
PXR_human           FLKIMAMLTELRSINAQHTQRLLRIQD---IHPFATPLMQELFGITG-------------
VDR_human           YAKMIQKLADLRSLNEEHSKQYRCLSFQPECSMKLTPLVLEVFGNEI-------------
FXR_human           FACLLGRLTELRTFNHHHAEMLMSWRV---NDHKFTPLLCEIWDVQ--------------
LXR_human           FPRMLMKLVSLRTLSSVHSEQVFALRL---QDKKLPPLLSEIWDVHE-------------


CAR_human           ------------------------------------------------------------
TSH_human           GEAGSLQGPVLQHQSPKSPQQRLLELLHRSGILHARAVCGEDDSSEADSPSSSEEEPEVC
PPARα_human         ------------------------------------------------------------
PPARγ_human         ------------------------------------------------------------
RXR_human           ----QMT-----------------------------------------------------
PXR_human           ----S-------------------------------------------------------
VDR_human           ----S-------------------------------------------------------
FXR_human           ------------------------------------------------------------
LXR_human           ------------------------------------------------------------


CAR_human           ----------
TSH_human           EDLAGNAASP
PPARα_human         ----------
PPARγ_human         ----------
RXR_human           ----------
PXR_human           ----------
VDR_human           ----------
FXR_human           ----------
LXR_human           ----------
```

Supplementary Figure S5: Immunoblots showing expression of tagged proteins.

A

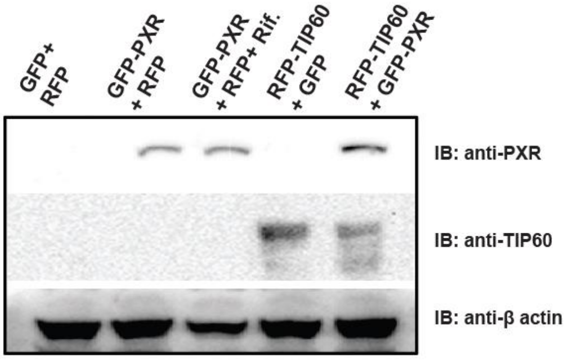

B

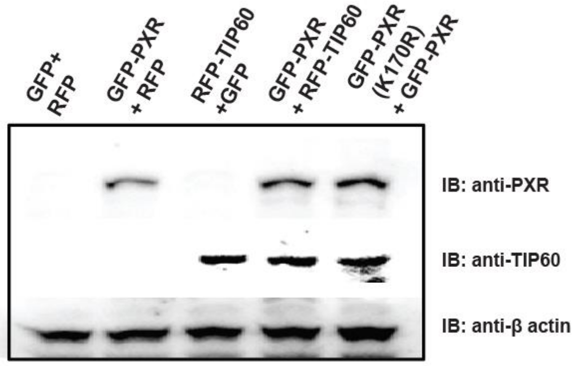

C

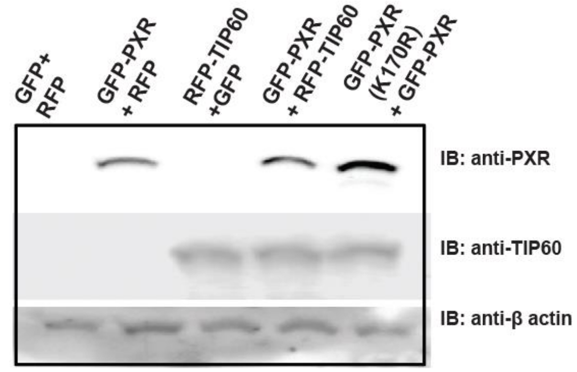

D

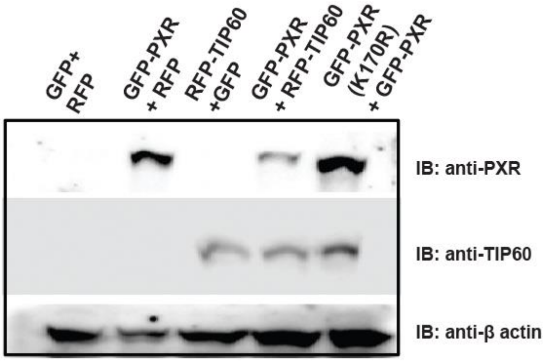

E

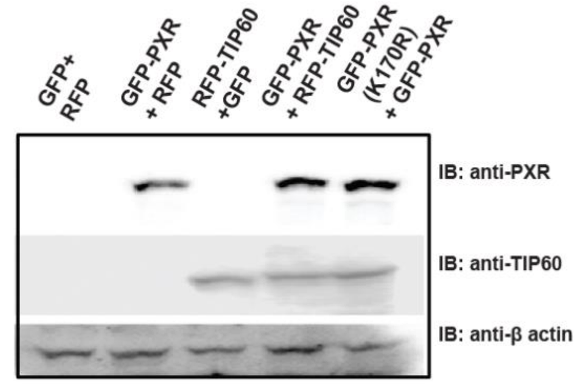

Supplementary Figure S6: Full-length immunoblots showing expression of proteins.

A

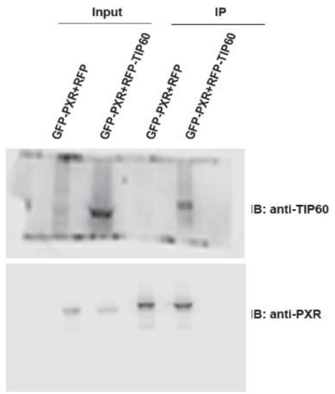

B

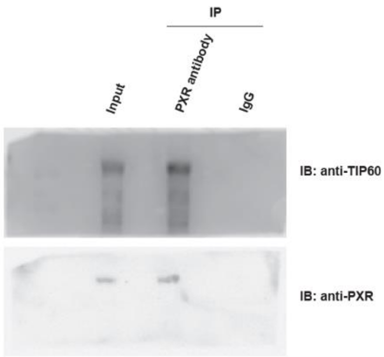

C

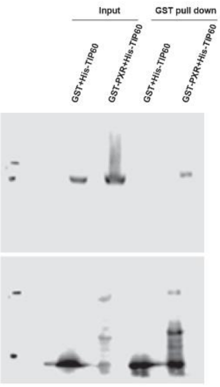

D

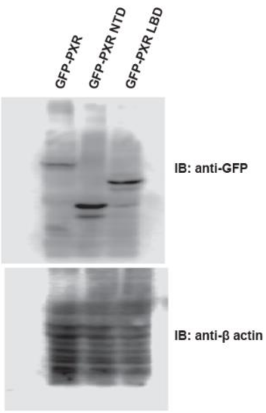

E

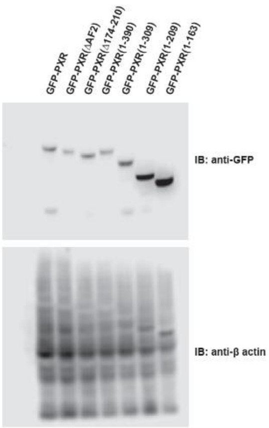

F

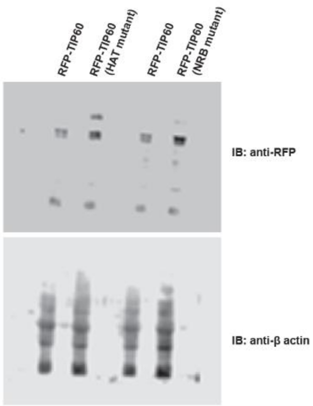

G

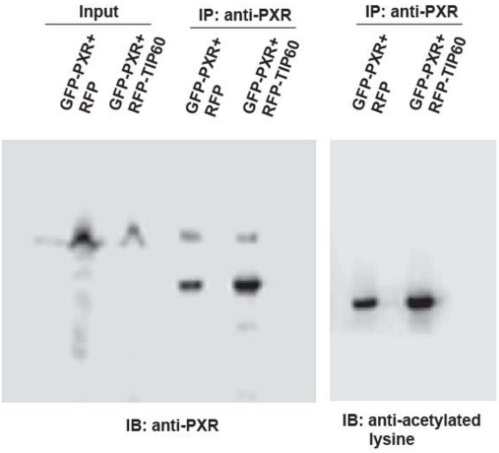

H

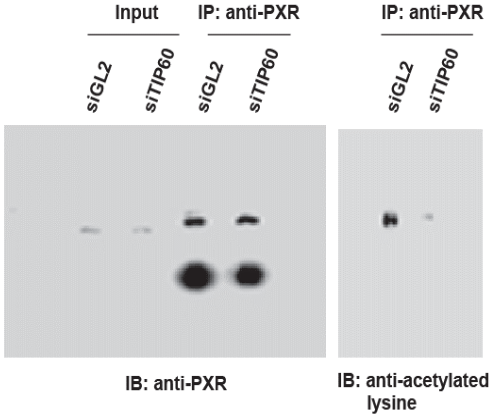

I

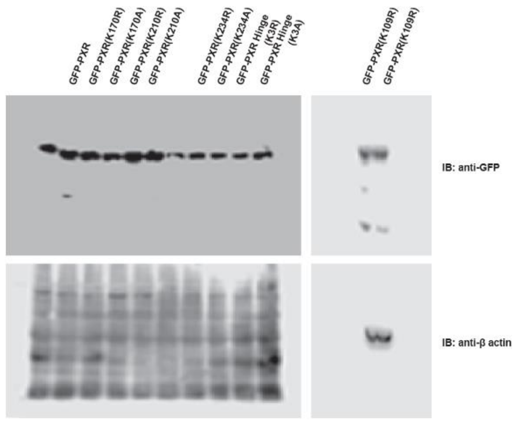

J

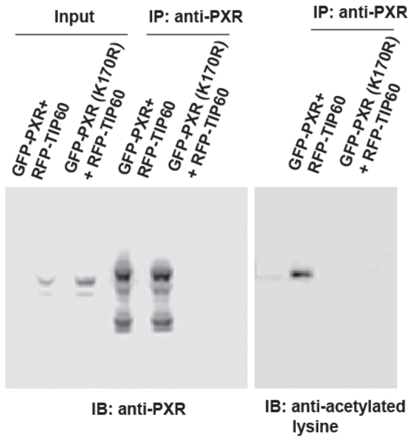

K

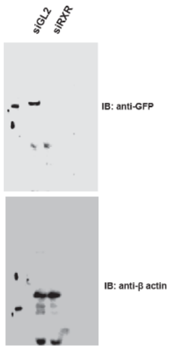

L

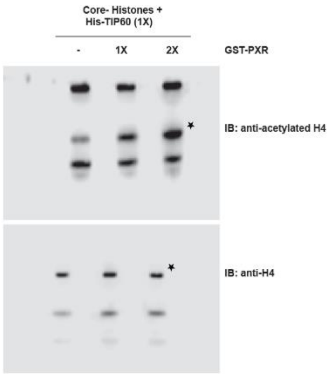

Supplementary Table 1: List of primers used in the study.

S.No.	Primer Name	Sequence (5' to 3')
1	hPXR(K109R)Fw	GAGCGGCATGAAGAGGGAGATGATCATG
2	hPXR(K109R)Rv	CATGATCATCTCCCTCTTCATGCCGCTC
3	hPXR(164-210)del Fw	ATGAAAACCTTTGACGTCTCTCTGCAGCTGCGG
4	hPXR(164-210)del Rv	ATGAAAACCTTTGACGTCTCTCTGCAGCTGCGG
5	hPXR(174-210)del Fw	TTCAAGAATTTCCGGGTCTCTCTGCAGCTGCGG
6	hPXR(174-210)del Rv	CTGCAGAGAGACCCGGAAATTCTTGAAAT
7	hPXRLBDdel1BamHIRv	CGGGATCCTCAAAAGGTTTTCATCTGAGCGTC
8	hPXRLBDdel2BamHIRv	CGGGATCCTCAAGAGCACAGATCTTTCCGGA
9	hPXRLBDdel3BamHIRv	CGGGATCCTCACAAGCAGTAGGACAGCCG
10	hPXRLBDdel4BamHIRv	CGGGATCCTCACAAGAACCTATGAGCAGGCT
11	hPXRFwKpnI	GGGGTACCATGCTGGAGGTGAGACCCA
12	hTIP60RvEcoRI	CGGAATTCCCACTTCCCCCTCTTGCT
13	hPXRdelAF2RvBamHI	CGGGATCCTCACGTAGCAAAGGGGTGT

14	hPXRFwBamHI	CGGGATCCATGCTGGAGGTGAGACCCA
15	hPXRRvEcoRI	CGGAATTCTCAGCTACCTGTGATGCCG
16	hTIP60HATmutFw	ACCCTGCCTCCCTACGAGCGCCGGGAGTACGGCAAG
17	hTIP60HATmutRv	CAGCAGCTTGCCGTACTCCCGGCGCTCGTAGGGAGG
18	hPXRNLSmutK66Fw	TGCAAGGGCTTTTTCGCGGCGGCCATGAAACG
19	hPXRNLSmutK66Rv	GTTGCGTTTCATGGCCGCCGCGAAAAAGC
20	hPXRNLSmutK91Fw	ATCACCCGGAAGACCGCGGCACAGTGCCAG
21	hPXRNLSmutK91Rv	GCAGGCCTGGCACTGTGCCGCGGTCTTCCG
22	HsPXRNtermFwEcoRI	CGGAATTCATGCTGGAGGTGAGACCCA
23	HsPXRNtermRvBamHI	CGGGATCCTCAACTTTTCTTCCGCTTGATC
24	HsPXRLBDFwEcoRI	CGGAATTCATGACAGAGGAGCAGCGGA
25	HsPXRLBDRvBamHI	CGGGATCCTCAGCTACCTGTGATGCCG
26	hPXRHingeK3RmutFw	AGGCGGGCCTTGATCAGGCGGAGGAGGAGTGAACG
27	hPXRHingeK3RmutRv	CCCTGTCCGTTCACTCCTCCTCCGCCTGATCAAGG
28	hPXRK170RmutFw	ACCTTCTCCCATTTCAGGAATTTCCGG
29	hPXRK170RmutRv	TGGCAGCCGGAAATTCCTGAAATGGGA
30	hPXRK210RmutFw	GATCTGTGCTCTTTGAGGGTCTCTCTG
31	hPXRK210RmutRv	CAGCTGCAGAGAGACCCTCAAAGAGCA
32	hPXRK234RmutFw	GCCGACAGTGGCGGGAGGGAGATCTTC
33	hPXRK234RmutRv	CAGGGAGAAGATCTCCCTCCCGCCACT
34	hPXRHingeK3AmutFw	AGGCGGGCCTTGATCGCGCGGGCGGCAAGTGAACG
35	hPXRHingeK3AmutRv	CCCTGTCCGTTCACTTGCCGCCCGCGCGATCAAGG
36	hPXRK170AmutFw	ACCTTCTCCCATTTCGCGAATTTCCGG
37	hPXRK170AmutRv	TGGCAGCCGGAAATTCGCGAAATGGGA
38	hPXRK210AmutFw	GATCTGTGCTCTTTGGCGGTCTCTCTG
39	hPXRK210AmutRv	CAGCTGCAGAGAGACCGCCAAAGAGCA
40	hPXRK234AmutFw	GCCGACAGTGGCGGGGCAGAGATCTTC
41	hPXRK234AmutRv	CAGGGAGAAGATCTCTGCCCCGCCACT
42	hTIP60FwBamHI	CGGGATCCATGGCGGAGGTGGGGGAG
43	hTIP60NRBmutRvEcoRI	CGGAATTCTCACCACTTCCCCCTCTTGCTCCAGTCCTTGGGAGT[illegible]<br>GTGCAGACACTTGGAGTCGATCCGCGCGGCCCGCTTGAGCAT
44	hRXRFw	CGGAATTCATGGACACCAAACATTTCCTG
45	hRXRRv	GGGGTACCAGTCATTTGGTGCGGCG
